# Data Resource Profile: The Lithuanian health data reuse pathway

**DOI:** 10.1093/ije/dyaf118

**Published:** 2025-07-06

**Authors:** Julius Juodakis, Simona Zubavičiūtė, Austėja Dapkutė, Saulė Greičienė, Rytis Masiliūnas, Indrė Narkevičienė, Justas Trinkūnas, Julija Žemaitytė, Gita Literskė

**Affiliations:** Department of Obstetrics and Gynecology, Institute of Clinical Sciences, University of Gothenburg, Gothenburg, Sweden; State Data Governance Group, State Data Agency, Vilnius, Lithuania; Centre for Digital Medicine, Translational Health Research Institute, Faculty of Medicine, Vilnius University, Vilnius, Lithuania; State Data Governance Group, State Data Agency, Vilnius, Lithuania; Clinic of Neurology and Neurosurgery, Institute of Clinical Medicine, Faculty of Medicine, Vilnius University, Vilnius, Lithuania; State Data Governance Group, State Data Agency, Vilnius, Lithuania; Clinic of Neurology and Neurosurgery, Institute of Clinical Medicine, Faculty of Medicine, Vilnius University, Vilnius, Lithuania; State Data Governance Group, State Data Agency, Vilnius, Lithuania; Centre for Digital Medicine, Translational Health Research Institute, Faculty of Medicine, Vilnius University, Vilnius, Lithuania; Department of Information Systems, Faculty of Fundamental Sciences, Vilnius Gediminas Technical University, Vilnius, Lithuania; State Data Governance Group, State Data Agency, Vilnius, Lithuania; State Data Governance Group, State Data Agency, Vilnius, Lithuania

Key Messages

- In 2022, Lithuania adopted the Law for Health Data Reuse, established a secure platform for remote analysis, and incorporated multiple data holders into a national data lake, creating a pathway for accessing all national health data for secondary use.
- The pathway can be used by researchers, public institutions, and private companies alike, to access data and use it in areas of public interest, such as research, development of drugs, or improving medical services.
- The available data include national-scale electronic health records, such as the Electronic Health Services and Cooperation Infrastructure Information System (ESPBI IS) and National Health Insurance Fund (NHIF) “Sveidra” systems, which currently contain >700 million recorded diagnoses, >100 million prescriptions and other information for 3.7 million patients.
- The data are highly representative and covers 90%–100% of the population. Data from individual hospitals, primary care providers, and other registries are also available and are linked to other administrative information, such as patients’ income, education, social benefits, etc. Most datasets are updated daily.
- The data can be accessed by applying to the State Data Agency.

## Data resource basics

### Lithuania: demographics and background

Lithuania is a Baltic region country of nearly 3 million people. Since regaining independence in 1990, Lithuania has undergone a period of rapid economic growth, reformed its healthcare, education, and other systems, and in 2004 joined the European Union (EU) and the North Atlantic Treaty Organization (NATO). Geographically, the country is relatively homogeneous. Ethnically, 85% of the residents identify as Lithuanian, with Polish, Russian, Belarussian, and Ukrainian minorities adding up to another 13%, largely concentrated in the eastern part of the country [1]. The administration, including healthcare, is centralized. The main cities, in particular Vilnius, are stable or growing in population, while rural area population is rapidly shrinking [1].

In numbers, Lithuania features one of the fastest growing economies in the Organization for Economic Cooperation and Development (OECD) [2], a gender equality score among the 10 highest in the world [3], 58% of young adults with tertiary education [4], but also substantial income inequality, comparable to that of the UK or USA [5], large avoidable mortality [2], and relatively low life expectancy, particularly for men (69.5 years, 78.8 for women; [6]).

Nonetheless, various demographic and healthcare issues that have been particularly prevalent are being tackled. Over the last decade, Lithuania has been the only state in the EU to reduce the numbers of traffic deaths by 50% [7]; alcohol consumption per capita decreased by 25% [1], emigration by about a third [1], suicide rate around two-fold [8, 9], and tuberculosis incidence more than two-fold [10]. Thus, Lithuanian health data can be a valuable source for studying the local policies and trends, in addition to standard epidemiological research applications.

### Healthcare in Lithuania

The Lithuanian healthcare system is universal and predominantly state-funded, with the majority of care delivered by public healthcare providers. However, the private sector is prominent in primary and dental care, and emerging in specialist services: in 2022, 8.4 million doctor visits were reported by private providers (of the 23.4 million total in Lithuania; [11]). The public sector includes 79 hospitals and 125 outpatient clinics versus approximately 16 and 694 respective institutions in the private sector [11].

The funding is predominantly managed by the National Health Insurance Fund (NHIF) under the Ministry of Health implementing a single-payer system. Other healthcare financing resources include the state budget, private expenses, and private insurance contributions. The national insurance covers all Lithuanian permanent residents, as well as certain groups of temporary and non-residents, paying compulsory health insurance contributions [12].

For the insured, primary and secondary care are free without any copay, except for certain services considered elective, such as cosmetic surgeries [13]. In private providers, only some of the services are covered by the NHIF. Emergency care is free for all permanent residents and certain other groups [12]. Different pharmaceuticals are covered to varying degrees between 0% and 100%; most medications for chronic diseases are fully covered.

Long waiting times are a major concern with public providers, though comparisons with other countries are unclear. Despite relatively low state spending (6.5% of gross domestic product), an OECD assessment in 2018 found that access to healthcare in Lithuania was generally good [2].

### Law for health data reuse

Large amounts of data are collected in the healthcare sector, and in 2022, Lithuania adopted the Law for Health Data Reuse [14]. The law supports the use of health data for scientific research, innovation in healthcare and development of treatments and services by establishing a single common pathway for accessing health data. The framework is similar to that used in existing cohorts and national resources, such as the Findata service in Finland [15], and the upcoming EU Regulation on the European Health Data Space [16]. Briefly, prospective applicants submit a description of the data needed and planned analysis to the State Data Agency (an office of the Lithuanian government, responsible for national statistics and data governance). The Agency evaluates the application, ensures that the requested data are appropriate for the stated aims, grants a permit, retrieves, links, de-personalizes, and provides the data to the applicant for analysis.

One of its key features is the ability to access a broad spectrum of data, beyond predefined cohorts or specific registries, allowing users to request nearly any health-related data from the country’s extensive electronic health records, national registries, and healthcare institutions. Different resources are linked on demand, unambiguously, through the national identification number (Lith. *asmens kodas*) issued to all citizens and residents [17]. While individuals cannot generally opt-out of the reuse of their data under this law, the data are de-identified before provision and cannot be traced back to specific individuals by the user. This approach aims to balance the need for detailed and flexible data analysis with strong privacy protections, ensuring compliance with the General Data Protection Regulation and strengthening public trust in the digital health system.

## Data collected

Lithuania collects national-coverage healthcare data in two large databases of health records, as well as a number of smaller registers and hospital information systems.

**Figure 1.**
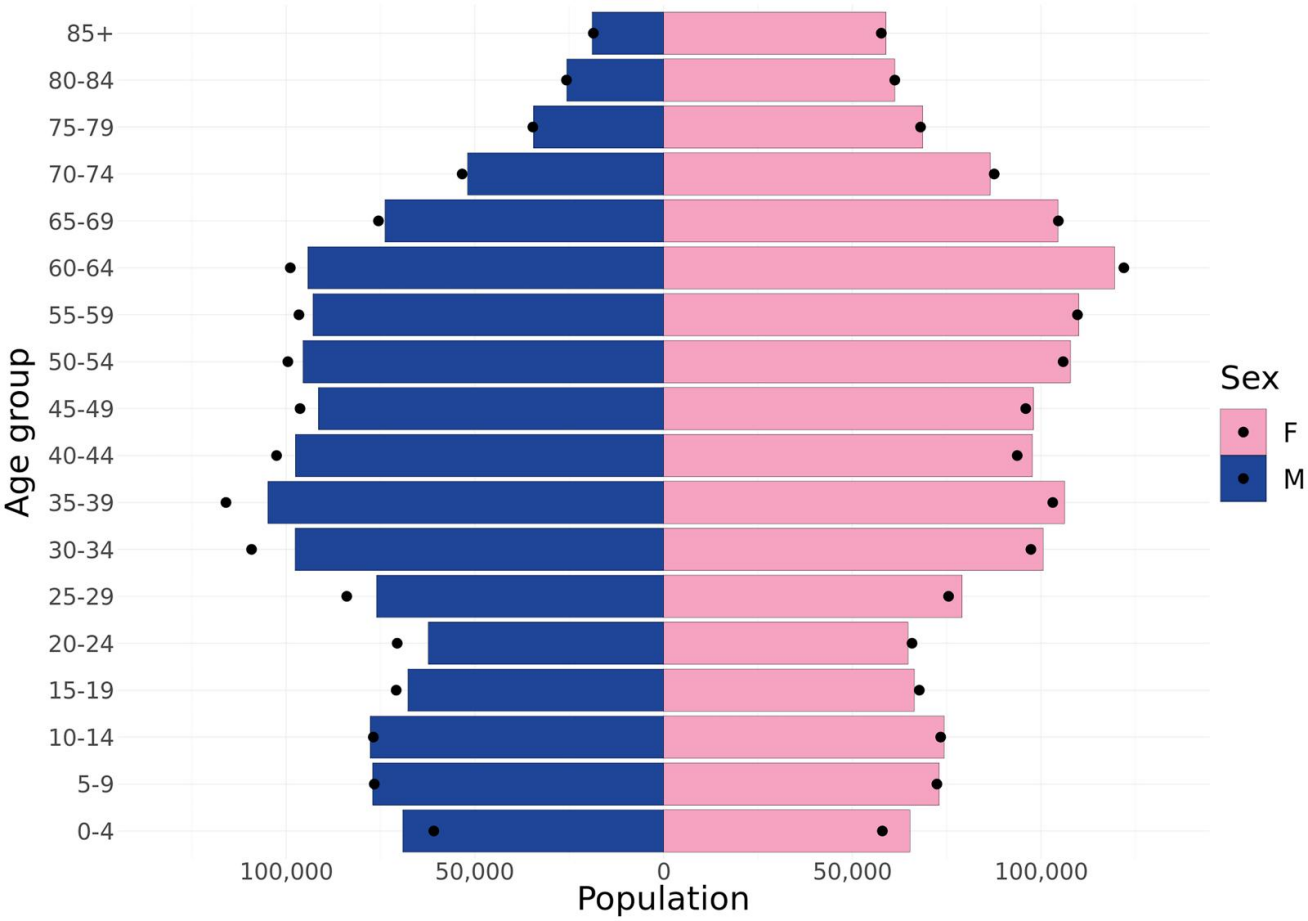
Age and sex distribution of outpatients in the ESPBI IS (national electronic health record system) and the general population of Lithuania for the year 2024. The bars represent the number of unique outpatients in each age group at the time of diagnosis. The black dots indicate the corresponding number of individuals in the general resident population of Lithuania. Infants may receive health records before getting a stable personal code and therefore may appear overrepresented. F: female; M: male.

The Electronic Health Services and Cooperation Infrastructure Information System (Lith. *Elektroninė sveikatos paslaugu ir bendradarbiavimo infrastruktūros informacinė sistema*, ESPBI IS) is a digital platform intended for all data collected during healthcare, by all providers—reporting inpatient and outpatient visits, referrals, diagnostic reports, drug prescriptions, births, vaccinations, and more. Currently (as of February 2025), ESPBI IS contains ∼357 million ICD-10-AM (Australian modification) coded diagnoses and procedures from 126 million outpatient visits for 3.7 million unique patients. Almost the entire population is represented in this data (Fig. 1). However, most finer clinical details are recorded only in unstructured text, which requires complex data analysis tools.

The health insurance system “Sveidra” collects detailed records from all healthcare providers receiving payments from the NHIF. This administrative data system has clear coding rules and well-structured data of procedures, diagnoses, medications, and their reference insurance prices. The system primarily contains data concerning state-funded medical services since its primary function is to record national insurance claims. Currently, “Sveidra” contains ∼741 million ICD-10-AM coded diagnoses from outpatient visits for 3.7 million unique patients.

In addition, a trove of data is still contained within hospital and outpatient clinic information systems, where each healthcare provider has the authority to determine what data to gather and how to organize it. This includes millions of laboratory test results, pathology reports, medical images, etc. Some crucial clinical characteristics, such as the location and stage of tumors, are only available from specialized disease registers, or provider systems which may be fragmented and heterogeneous.

A non-exhaustive list of the main health data sources is provided in Table 1. The State Data Agency also holds data from other areas besides healthcare, currently totalling 621 information systems and registries [18], and is cataloguing all 1400+ data sources of the Lithuanian public sector from which data can be retrieved on demand. Much of this data are at the individual level and can be linked securely and consistently through the patient’s personal code. A list of major information systems and registries can be found at https://registrai.lt. Admittedly, some data are still in silos such as datasets collected by individual research groups or those held by private institutions. The Law for Health Data Reuse pathway allows researchers to request and analyze combined data from all these sources at the (de-identified) subject level.

**Table 1.** A non-exhaustive list of main data sources relevant to health research in Lithuania and available through the Law for Health Data Reuse

Source	Time frame covered	Objects of record	Key features recorded
ESPBI IS	Introduced in the end of 2015; mandatory usage in many areas since 2018	All records of inpatient and outpatient visits, referrals, vaccinations, for individuals receiving healthcare in Lithuania	ICD-10-AM coded health conditions; unstructured case description; visit date, location, healthcare specialist
ESPBI IS ePrescriptions	Introduced in the end of 2015; mandatory since 2018	All prescriptions issued in Lithuania	ATC code, prescribed amount, dates of prescription and dispensation, dosage, expected supply end date
NHIF “Sveidra”	Since 2014	Any medical procedures covered by the NHIF, also some non-covered procedures; all NHIF-covered prescriptions, plus centrally purchased pharmaceuticals and devices	Procedure ACHI codes; ICD-10-AM coded health conditions accompanying procedures; start and end dates of inpatient stays; dispensation dates; medications and medical devices purchased centrally; insurance status of the patients
Causes of Death Registry	Since 2010	All deaths in Lithuania and all deaths of Lithuanian citizens abroad	ICD-10-AM coded causes of death (immediate, intermediate and underlying); type of death (e.g. suicide, accident); date and location
Cancer Registry	Since 1978	Initial diagnosis of every cancer case diagnosed in Lithuania	ICD-10-AM coded diagnoses; tumor grade, stage, TNM classification, morphology, topography (at first diagnosis)
MedVAIS	Since 2015	Ultrasound, CT, MRI, EEG, ECG images	Medical images and/or descriptions
Care provider EHR systems	Varies between providers	All patients receiving care	All structured and unstructured data collected during care, including medical history, diagnoses, treatments, procedures, test results, and medications; structure and standards vary between providers
Population Register	Main events since 19th century, residential addresses since approx. 1993	Lithuanian citizens; all persons registered as living in Lithuania or receiving civil documents in Lithuania	Births; deaths; ethnicity; nationality; civil status; registration address
State Social Insurance Fund Board (“SoDra”)	Payments since approx. 2006	All persons employed by companies registered in Lithuania or receiving any welfare in Lithuania	Various data on employment and welfare including total salary, sickness benefits with accompanying reason for the claim (ICD-coded)

ACHI: Australian Classification of Health Interventions; ATC: Anatomical Therapeutic Chemical; CT: computed tomography; ECG: electrocardiogram; EEG: electroencephalogram; EHR: Electronic Health Records; ESPBI IS: Electronic Health Services and Cooperation Infrastructure Information System; ICD-10-AM: International Statistical Classification of Diseases and Related Health Problems, Tenth Revision, Australian Modification; MRI: magnetic resonance imaging; NHIF: National Health Insurance Fund; TNM: TNM Classification of Malignant Tumors.

## Data resource use

As of January 2025, 25 permits have been issued for data usage following the Health Data Reuse law. Since this is a recent process, most of these projects are still in progress and are yet to be published; also, around half of the projects are not research-oriented, e.g. internal reports or models. The first publication using this data was recently published [19].

To illustrate the possibilities within the linkage of different source data, we present two small examples. These should not be interpreted as finished analyses but only as pilot demonstrations of the possibilities within the Lithuanian health data reuse.

### Tick-borne encephalitis mapping

**Figure 2.**
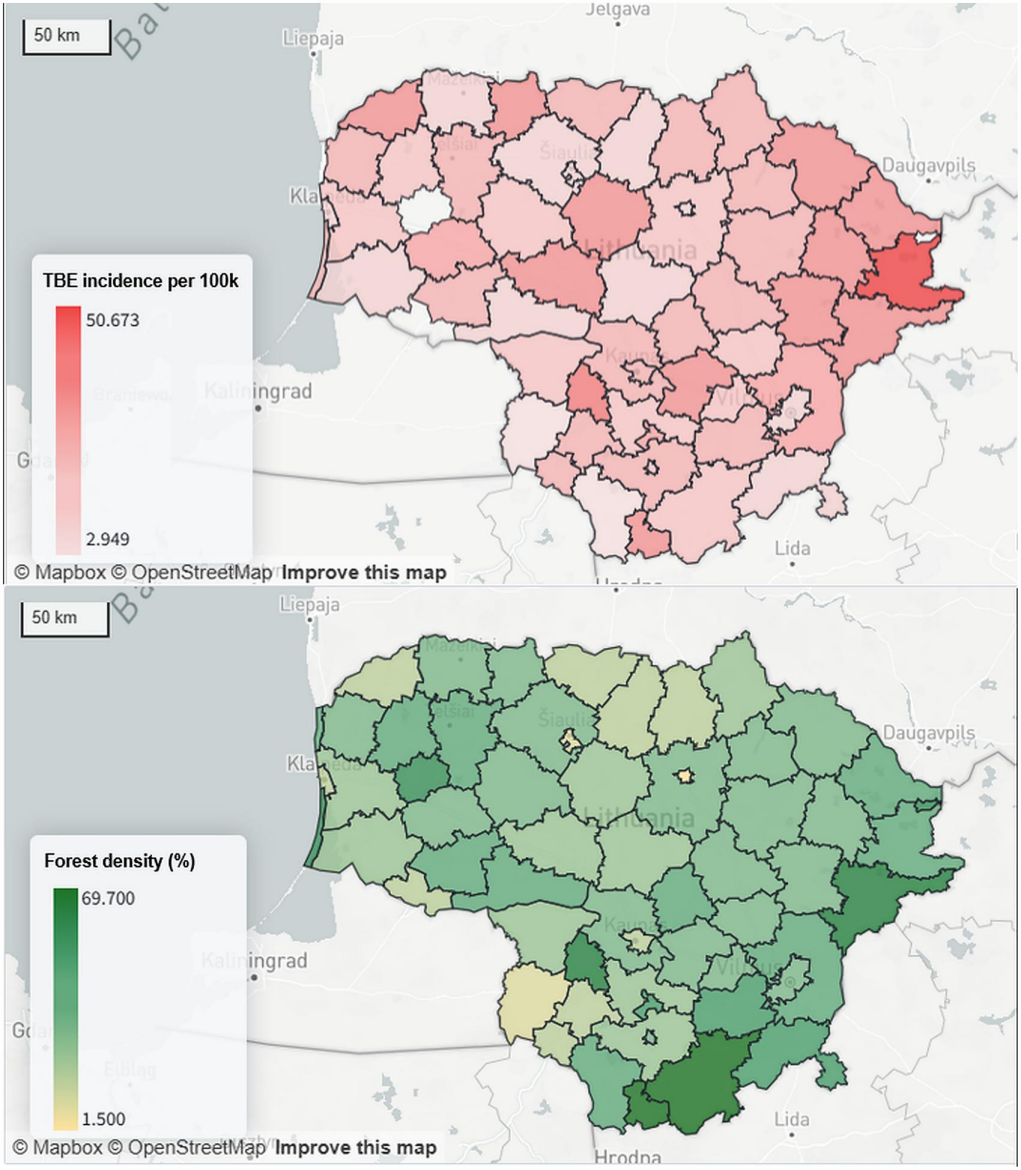
The percentage of forest land in Lithuania’s municipalities for the year 2024 (bottom). The incidence of tick-borne encephalitis (TBE) per capita in Lithuania’s municipalities for the year 2024 (top). A person is considered a TBE case if they received three or more A84.X (where X—any number) entries in the ESPBI IS (national electronic health record system) over 2024. The patient’s residential address at the time of first TBE diagnosis in 2024 was used to calculate the TBE incidence per capita in Lithuania’s municipalities. Analysis was performed in Palantir Foundry platform.

Lithuania is a highly endemic country for tick-borne encephalitis (TBE) [20], an infectious disease that affects the central nervous system and is transmitted through the bite of infected ticks, which are commonly found in areas with higher forest density. Using TBE diagnosis records from ESPBI IS, patient residential addresses from the population register, and forest registry, we created maps of TBE incidence and forest density across the 60 Lithuania’s municipalities (Fig. 2).

While the greatest forest density is in the southeastern region of the country, patients infected with TBE lived mostly in the eastern area but generally were uniformly distributed. This phenomenon can be interpreted in various ways: ticks that transmit TBE are mostly active in the warmer seasons, which may correspond with vacation or other activities away from home for many people. Moreover, a smaller forest with high human activity might see a higher incidence of TBE due to increased exposure to ticks, while a very dense forest with fewer human visitors may have smaller risk even if it has a large tick population. Finally, a larger fraction of the population in forested areas may be vaccinated against TBE. Researchers interested in this question could use the Law for Health Data Reuse to analyze factors such as the timing of diagnosis (e.g. summer versus winter months), vaccination rates, historical temperatures, and other variables.

### Cancer rates by occupation

In the second example, we evaluated the incidence of cancer diagnoses among occupational groups in Lithuania. This example uses employer-reported occupation for each person and diagnosis records for each patient from the ESPBI IS. These two databases are then linked at the person level and aggregated.

The 10 highest-incidence and 10 lowest-incidence occupation groups are presented in Table 2. The findings suggest that the highest cancer rates are found among the occupational groups of “Managers in the field of aged care and social services” and “Janitors and workers in related professions,” while the lowest rates are observed in the groups of “Trade brokers” and “Web and multimedia developers.” These findings are likely explained by differences in individuals’ age in different occupations: employees in more senior positions are likely older, which contributes to a greater hazard of developing cancer in the immediate future. Conversely, web developer and trade broker groups are likely to consist of younger individuals and thus have a lower cancer risk. A proper incidence analysis should incorporate this and also assess a full career history, the patients’ comorbidities, and other factors which are available in the data supplied by the State Data Agency.

**Table 2.** Incidence of cancer diagnosis among occupational groups in Lithuania (10 highest-incidence and 10 lowest-incidence groups shown)^a^

Occupation	Incidence per 100 persons since 1 January 2023
Highest-incidence 10	
Managers in the field of elderly care and social services	7.89
Sweepers and related laborers	7.25
Hotel managers	7.09
Legislators	6.91
Building caretakers	6.77
Bus and trolleybus drivers	6.72
Social work and counseling professionals	6.71
Managers of special-interest organizations	6.54
Senior cleaning and general maintenance workers in offices, hotels and other establishments	6.45
Managers in the field of healthcare services	6.23
Lowest-incidence 10	
Credit and loans officers	0.99
Application developers	0.98
Air traffic controllers	0.93
Software developers	0.92
Aircraft engine mechanics and repairers	0.86
Draftsmen	0.81
Waiters	0.76
Aircraft pilots and other specialists in related professions	0.68
Web and multimedia developers	0.42
Trade brokers	0.00

a The occupation(s) for each worker in Lithuania were determined as of 1 January 2023, based on employer reports and the Lithuanian classification of occupations (unit-group level). We count persons as having cancer if they received three or more cancer-related diagnosis entries (ICD-10-AM C codes) in the Lithuanian ESPBI IS after 1 January 2023. In cases where an individual held multiple occupations, all were included in the analysis. Only occupations with > 100 valid workers were included.

## Strengths and weaknesses

### Strengths

The Lithuanian data ecosystem provides unique opportunities for data reuse. The main databases provide comprehensive, centralized, national-scale coverage of essential healthcare records, including outpatient and inpatient visits, referrals, drug prescriptions, and medical devices. In addition to these, many smaller, specialized information systems and registries in diverse areas are available and can be reliably linked under the framework established by the Law for Health Data Reuse. Thus, with the wide and increasing range of data on socio-economic factors (e.g. education level, employment, income), mortality, lifestyle (e.g. drug usage through admissions to addiction centers), crime (e.g. traffic offenses), parent–child linkages, and geography, all available from the State Data Agency, a variety of deeper, multi-faceted research questions can be answered. Healthcare providers, policymakers, researchers, also companies and even private individuals can access these datasets on an equal basis, to monitor healthcare trends, evaluate the effectiveness of interventions, and otherwise drive enhancements in patient outcomes.

### Weaknesses

First, Lithuanian healthcare data possess a relatively short historical span (with an exception of the Cancer Registry, which was established in 1978). Data from the NHIF “Sveidra” database are available from 2014, mandatory usage of the ESPBI IS commenced only in 2018, and the development of electronic health record systems in most clinics also started around this period. At the moment, this can limit various longitudinal or rare disease studies, although statistical modeling tools can be used to mitigate this [21, 22].

Despite high adoption of the ESPBI IS, the integration between different electronic health record systems is still a challenge. Providers submit some of the data to ESPBI IS directly; however, most of the collected data are stored locally, in varying formats, standards, and coverage. While some data (such as the prescriptions) are highly structured, much of the medically important information is recorded in free text, which is difficult to de-identify and is of limited use for analysis. The State Data Agency has already initiated pilot studies to address this by introducing additional structured fields and by parsing existing records using large language models. Similarly, data of other modalities, such as medical images, will also require advanced processing, and thus higher time and cost, to provide. Note also that researchers can merge the administrative databases with data that they collected based on consent, such as clinical trial records or patient-reported outcomes, to create finely-phenotyped datasets for their particular area of interest.

While administrative data sources allow much larger samples, such records may not be as accurate as those collected for clinical trials or specific research projects. The Cancer Registry and Causes of Death Registry are maintained by experts who extract relevant data from administrative sources and validate it; therefore, they are likely highly accurate. However, we are not aware of any publications quantitatively evaluating the accuracy of administrative records in these or other areas.

Although almost any dataset from any system can be requested on-demand, the lack of a single catalog of pre-formed datasets that can be ordered has been a significant barrier. However, a major project is underway that aims, by mid-2026, to create a complete catalog of all Lithuanian data in the public sector and to ensure that it is easy to access and analyze.

## Data resource access

The data resources presented here are accessible for secondary use through the Lithuanian State Data Agency. Individual researchers, as well as national and private institutions, can request data needed for their study through an online form at the Lithuanian Official Statistics Portal [23]. Data access follows a five-step procedure: (i) researchers plan their study, identify what data sources, features, and selection criteria would be applicable to answer their questions; (ii) researchers fill out the initial application form; (iii) State Data Agency evaluates the feasibility of the proposal, consults on the study design, and together with the applicant produces a final specification of data to be extracted; (iv) relevant fees are calculated and paid, and a permit is issued; (v) the requested data are collected, pseudonymized, and provided in a secure environment, accessible only to the specified researchers [14]. Data are generally provided as de-identified patient-level records, although some further anonymization may be used in particularly sensitive cases.

Currently (January 2025), the primary applicant must operate in Lithuania. Foreign analysts may be included by an agreement with the primary applicant. Under the European Health Data Space regulation, expected to come into force soon, applicants from all EU states will be eligible to apply on an equal basis. Nonetheless, the State Data Agency recommends all applicants to collaborate with local experts to ensure understanding of the data collection nuances. The State Data Agency does not have any requirements for co-authorship and strongly recommends following the ICMJE criteria.

Data are provided in the State Health Data Platform, a secure environment for processing, based on the Palantir Foundry platform. Researchers access this environment via browser and can analyze data using Python, R, or no-code analysis tools. In exceptional cases, it is possible to download data to another environment, provided that strict security protocols, data protection measures, and legal requirements are met.

## Ethics approval

The present study does not include novel biomedical research and therefore does not require an ethics approval as defined under the national law.

## Data Availability

See "Data resource access" above.
